# Metabolic engineering of cereal lipids: from omega-3 fatty acids to wax esters and pheromones

**DOI:** 10.1007/s44307-026-00124-9

**Published:** 2026-07-24

**Authors:** Meng-Tian Li, Jia-Ting Lin, Pedro García-Caparros, Li-Hua Zhu, Nan Yao, Yi-Han Xia

**Affiliations:** 1 Guangdong Provincial Key Laboratory of Plant Stress Biology, State Key Laboratory of Biocontrol, School of Agriculture and Biotechnology, Sun Yat-Sen University, Shenzhen, 518107 People’s Republic of China; 2 School of Life Sciences, Sun Yat-sen University, Shenzhen, 510275 People’s Republic of China; 3 Department of Superior School Engineering, University of Almería, Almería, Spain; 4 Department of Plant Breeding, Swedish University of Agricultural Sciences, Lomma, Sweden

**Keywords:** Cereal, Lipid metabolism, PUFAs, Wax esters, Insect pheromones

## Abstract

Cereals are emerging as attractive platforms for the sustainable production of high-value lipids through metabolic engineering. Although plant lipids play essential biological roles and have considerable economic value, their conventional production from natural sources is often limited by sustainability, scalability and cost. Recent advances in synthetic biology enable the reprogramming of seed lipid metabolism for the tailored synthesis of valuable lipid compounds. In this review, we first summarize the core pathways of fatty acid biosynthesis and triacylglycerol assembly in seeds, together with the genetic transformation and genome editing toolkits available for major cereals. We then highlight recent progress in the heterologous production of specialized lipids, including eicosapentaenoic acid (EPA), docosahexaenoic acid (DHA), wax esters, and insect sex pheromones, in engineered plant systems. Finally, we discuss the potential of cereals as scalable and sustainable platforms for the production of high-value lipids. Together, these advances position engineered cereals as promising plant-based factories for applications in agriculture, nutrition, and the emerging bio-based economy.

## Introduction

Lipids comprise a highly diverse class of hydrophobic or amphipathic biomolecules that play essential roles in membrane architecture, energy storage, signaling transduction, and adaptation to environmental change (Fahy et al. 2009; Li et al. 2016). In plants, fatty acids (FAs) serve as the primary building blocks of most lipid classes, including membrane lipids that form the structural matrix of biological membranes, triacylglycerols (TAGs) that accumulated in seeds, and cuticular waxes deposited on the surfaces of leaves and seed coats that regulate transpirational water loss and enhance protection against pathogens and abiotic stress (Li et al. 2016). In seeds, glycerolipids constitute the predominant lipid class and are broadly classified into neutral glycerides, including monoacylglycerols (MAGs), diacylglycerols (DAGs), triacylglycerols (TAGs), and polar glycerides, including glycerophospholipids and glycoglycerolipids, with TAGs representing the major storage form (Fahy et al. 2009). Variation in FA chain length, degree of saturation, and structural modification underpins the broad functional and physicochemical diversity of plant lipids. In addition to their indispensable biological functions, numerous specialized lipids possess substantial economic and industrial value. As many of these compounds can be derived from shared FA-based, metabolic frameworks, they have emerged as prominent targets for plant metabolic engineering. In this review, we highlight three representative product classes: 1) very-long-chain omega-3 polyunsaturated fatty acids (VLC-PUFAs), valued for their benefits to human nutrition; 2) plant- and insect-derived waxes, which have applications in cosmetics, pharmaceuticals, and other specialty sectors; 3) lepidopteran sex pheromones, which are used as environmentally benign tools for integrated pest management (Petkevicius et al. 2020; Domergue and Miklaszewska 2022).

PUFAs are FAs containing two or more double bonds. Among them, omega-3 and omega-6 FAs are classified as essential fatty acids (EFA) as they cannot be synthesized de novo in humans and must therefore be acquired through the diet (Vaezi et al. 2013). In omega-3 FAs, the first double bond is positioned between the third and fourth carbon atoms from the methyl end of the chain, whereas in omega-6 FAs it occurs between the sixth and seventh carbon atoms. Both omega-3 and omega-6 PUFAs are indispensable for human health, contributing to the regulation of inflammatory processes, cardiovascular function, and neural development (Venegas-Calerón et al. 2010). Current nutritional recommendations generally propose an omega-6 to omega-3 FAs ratio of approximately 4:1 to 1:1. However, modern diets in many populations are characterized by excessive omega-6 intake relative to omega-3 intake, therefore contributing to an unfavorable FA balance. The most nutritionally significant omega-3 PUFAs include α-linolenic acid (ALA; 18:3^∆9, 12, 15^), eicosapentaenoic acid (EPA; 20:5^Δ5, 8, 11, 14, 17^) and docosahexaenoic acid (DHA; 22:6^Δ4, 7, 10, 13, 16, 19^) (Vaezi et al. 2013). Because the endogenous conversion of ALA to EPA and DHA is highly inefficient in humans, and because direct dietary sources of EPA and DHA are limited, insufficient intake of these long-chain omega-3 FAs is widespread (Vaezi et al. 2013). Currently, dietary EPA and DHA are obtained primarily from marine fish and microalgae. However, reliance on marine resources raises concerns related to sustainability and supply security, highlighting the need for alternative and renewable sources of these PUFAs (Venegas-Calerón et al. 2010). In this regard, the engineering terrestrial plants for EPA and DHA production represents a particularly promising approach to enhance accessibility, improve nutritional security, and promote sustainable production systems.

Wax esters (WEs) are neutral lipids formed by the esterification of a fatty alcohol with a FA chain. Owning to their favorable physicochemical properties, WEs are widely used in lubricants, cosmetics, and specialty chemicals (Heilmann et al. 2012; Whitehead et al. 2025). Despite their considerable industrial importance, natural sources of WEs are limited and commercial supply remains constrained. Jojoba (*Simmondsia chinensis*) seed oil is the principal plant-derived source of WEs; however, its cultivation is restricted largely to arid and semi-arid regions. Historically, spermaceti obtained from sperm whales was also an important commercial source, but its use was ceased followed the 1986 international moratorium on commercial whaling. Although chemical synthesis offers an alternative means of production, it remains costly, energy-intensive and environmentally unsustainable (Heilmann et al. 2012; Whitehead et al. 2025). Together, these limitations underscore the need to develop scalable and sustainable crop-based platforms for WEs production.

About 75% of identified lepidopteran pheromones are lipids, collectively classified as type I sex pheromones. These molecules typically comprise C_10_–C_18_ fatty alcohols, acetates or aldehydes that mediate species-specific mate attraction (Ando et al. 2004). Recent studies have shown that insect pheromone biosynthetic enzymes can be rationally assembled and functionally expressed in plant hosts. By leveraging endogenous FA metabolic pathways, engineered plants can heterologously produce insect pheromone components, thereby offering a promising and environmentally sustainable approach for integrated pest management (Löfstedt and Xia 2021; Kallam et al. 2023).

The increasing demand for these high-value lipids, together with the limitations associated with conventional production sources, has stimulated interest in alternative and more sustainable production platforms (Hu et al. 2026; Napier 2026). Synthetic biology applies engineering principles to the design and redesign on biological systems. Over recent decades, the field has expanded rapidly, driven by advances in DNA synthesis, genome editing, and systems biology. In plants, synthetic metabolic engineering enables the introduction of genes from non-plant organisms, thereby establishing novel biosynthetic pathways and conferring the capacity to produce target compounds (Zhu et al. 2020). Plants have been genetically and metabolically engineered to produce valuable lipids (Haslam et al. 2016; Bates and Shockey 2025; Hu et al. 2026; Napier 2026). For example, the accumulation of EPA and DHA in the seeds of Arabidopsis, Camelina and canola was achieved through seed‐specific expression of a heterologous omega-3 VLC-PUFA biosynthetic pathway (Petrie et al. 2014, 2020; Han et al. 2022). This pathway comprises a suite of genes, predominantly derived from marine microorganisms, that convert the C_18_ FA precursors into omega-3 VLC-PUFAs (Petrie et al. 2014, 2020; Han et al. 2022). In addition, the heterologous production and accumulation of WEs and insect pheromones in plants have been successfully demonstrated (Petkevicius et al. 2020; Löfstedt and Xia 2021; Demski et al. 2022; Domergue and Miklaszewska 2022; Wang et al. 2022; Zhu et al. 2016; Iverson et al. 2017).

Among cultivated plants, cereals possess well-established agronomic systems, extensive cultivation areas, and mature supply chains, making them attractive chassis for industrial-scale production of beneficial lipids through metabolic engineering (Barthole et al. 2012; Slama et al., 2021; Mangla et al. 2025). To provide a comprehensive overview of recent advances in plant lipid metabolic engineering, we conducted a systematic literature search in the Web of Science database covering publications from 2010 to May 2026. Retrieved articles were screened for relevance to the topic, and additional studies were identified through citation tracking and examination of reference lists. This review summarizes lipid biosynthesis pathways in plant seeds and highlights key regulatory notes and metabolic engineering targets. We further assess recent progress in the heterologous production of omega-3 VLC-PUFAs, WEs, and moth sex pheromones in plants. Finally, we discuss the prospects for developing cereal-based “plant factories” as sustainable platforms for the bioproduction of high-value lipids.

## Seed lipid biosynthesis and its engineering in cereals

### Overview of lipid biosynthetic pathways in seeds

**Fig. 1:**
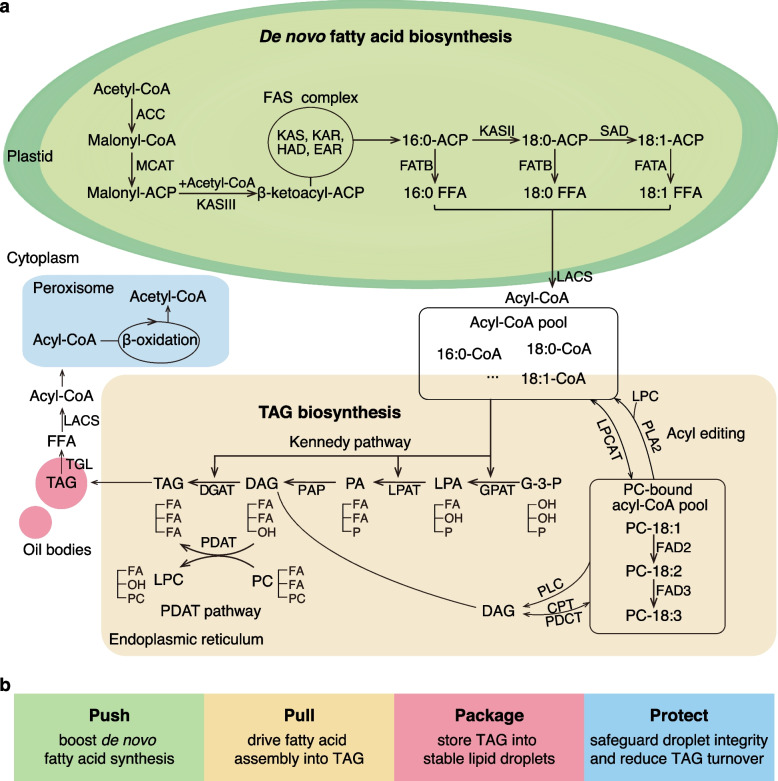
Overview of de novo FA and TAG biosynthesis pathways in seeds and strategies for lipid improvements. **a** Plants FA and TAG biosynthesis pathways. De novo FA biosynthesis occurs in the plastid and forms cytoplasmic acyl-CoA pool, after leaving the plastid and being added to CoA by LACS. TAG biosynthesis occurs in the endoplasmic reticulum via Kennedy pathway and PDAT pathway. The cycles of PC deacylation and LPC reacylation catalyzed by PLA_2_ and LPCAT, deliver acyl-CoAs between PC-bound acyl-CoA pool and acyl-CoA pool. The acyl and/or headgroup exchanges happen between PC and DAG via PLC, CPT and PDCT. TAGs package into oil bodies. FFAs released from TAG hydrolysis are activated by LACS or ACS and then degraded via β-oxidation pathway in the peroxisome. Abbreviation, ACC: acetyl-CoA carboxylase; ACP: acyl carrier protein; CoA: Coenzyme A; FFA: free fatty acid; CPT: choline phosphotransferase; DAG: diacylglycerol; DGAT: diacylglycerol acyltransferase; EAR: enoyl-ACP reductase; FATA/FATB: fatty acyl-ACP thioesterase A/B; GPAT: glycerol-3-phosphate acyltransferase; G-3-P: glycerol-3-phosphate; HAD: β-hydroxyacyl-ACP dehydratase; KAR: β-ketoacyl-ACP reductase; KAS: β-ketoacyl-acyl carrier protein synthase; LACS: long-chain acyl-CoA synthetase; LPA: lysophosphatidic acid; LPAT: lysophosphatidic acid acyltransferase; LPCAT: Acyl-CoA: lysophosphatidylcholine acyltransferase; MCAT: malonyl-CoA: acyl carrier protein transacylase; PA: phosphatidic acid; PAP: phosphatidic acid phosphatase; PDAT: phospholipid: diacylglycerol acyltransferase; PLA2: phospholipase A2; PC: phosphatidylcholine; PLC: phospholipase C; PDCT: phosphatidylcholine diacylglycerol cholinephosphotransferase; SAD: stearoyl-ACP desaturase; TAG: triacylglycerol. **b** Push, pull, package, protect strategies boost lipid contents by increasing FA synthesis in the plastid, driving FA assembly into TAG in the ER, storing TAG into stable cytoplasmic oil bodies, and protecting TAG stability and oil bodies integrity, respectively

Plant lipid biosynthesis occurs primarily in the plastid and endoplasmic reticulum (ER) (Li et al. 2016). The plastid-localized route is generally referred to as the prokaryotic pathway, whereas lipid assembly in the ER following export of FA precursors from plastids is commonly termed the eukaryotic pathway (Li et al. 2016; Vanhercke et al. 2019). In plants, de novo FA synthesis takes place in plastids. The resulting FAs are subsequently exported to the cytosol, where they enter the acyl-CoA pool and serve as substrates for TAG synthesis in the ER (Fig. 1a) (Vanhercke et al. 2019; Magdalena et al. 2021).

De novo FA synthesis begins with the formation of acetyl-CoA, which is primarily derived from pyruvate produced through glycolysis and the pentose phosphate pathway derived pyruvate via the plastidic pyruvate dehydrogenase complex. Acetyl-CoA is subsequently carboxylated by carboxylase (ACC) to produce malonyl-CoA (Vanhercke et al. 2019). Malonyl-CoA is then transferred to acyl carrier protein (ACP) by malonyl-CoA: ACP transacylase (MCAT), generating malonyl-ACP. β-ketoacyl-ACP synthase III (KAS III) catalyzes the initial condensation between acetyl-CoA and malonyl-ACP to form a C4 β-ketoacyl-ACP intermediate, which is subsequently converted to saturated C4:0-ACP through sequential reduction, dehydration, and reduction reactions. Repeated two-carbon elongation cycles catalyzed by the fatty acid synthase (FAS) complex then produce C16:0-ACP, which can be further elongated by KAS Ⅱ to C18:0-ACP, and subsequently desaturated to C18:1-ACP (Park et al. 2023). FAs are released from ACP by fatty acyl-ACP thioesterases (FATs) (Fig. 1a). In this process, saturated and unsaturated acyl-ACPs are hydrolyzed primarily by fatty acyl-ACP thioesterase B (FATB) and fatty acyl-ACP thioesterase A (FATA) respectively, generating free FAs (FFAs) that are subsequently exported from the plastid, possibly with the assistance of lipid transfer proteins (LTPs) (Wang et al. 2015). These saturated FAs (SFAs) may then undergo desaturation to produce unsaturated FAs (UFAs), including palmitoleic acid (C16:1^Δ9^), oleic acid (C18:1^Δ9^), linoleic acid (C18:2^Δ9, 12^) and α-linolenic acid (C18:3^Δ9, 12, 15^) (González-Thuillier et al. 2021). In higher plants, the most abundant FAs are typically C16:0 (palmitic acid), C18:0 (stearic acid), C18:1, C18:2, and C18:3 (Vanhercke et al. 2019; He et al. 2020).

Once exported from plastids, newly synthesized FFAs are activated to acyl-CoA esters by long-chain acyl-CoA synthetase (LACS) at the plastid envelope or in the cytosol, thereby contributing to the cytosolic acyl-CoA pool (Fig. 1a). These acyl-CoAs are subsequently delivered to the ER membrane, likely mediated via acyl-CoA binding proteins (ACBPs), where they serve as substrates for glycerolipid biosynthesis (Andolfo et al. 2015; Guo et al. 2019). TAG formation in plants occurs through two major pathways: the predominant acyl-CoA-dependent Kennedy pathway and the acyl-CoA-independent phospholipid: diacylglycerol acyltransferase (PDAT) pathway (Magdalena et al. 2021). In the Kennedy pathway, glycerol-3-phosphate (G-3-P) serves as the initial acyl acceptor. Sequential acylation reactions catalyzed by glycerol-3-phosphate acyltransferase (GPAT), lysophosphatidic acid acyltransferase (LPAT), and diacylglycerol acyltransferase (DGAT) incorporate three fatty acyl-CoA molecules to ultimately form TAG. In contrast, PDAT catalyzes the transfer of an acyl group from phospholipids, primarily phosphatidylcholine (PC), to DAG, thereby generating TAG and lysophosphatidylcholine (LPC) (Magdalena et al. 2021).

In addition to contributing to TAG synthesis via PDAT, PC functions as a central metabolic hub for PUFA synthesis and their subsequent incorporation into TAG through acyl editing (Fig. 1a). During acyl editing, FAs dynamically cycle between PC-bound form and the acyl-CoA pool through coordinated PC deacylation and LPC reacylation. PC deacylation can be catalyzed either by phospholipase A_2_ (PLA_2_) or through the reverse reaction of acyl-CoA: lysophosphatidylcholine acyltransferase (LPCAT) (Lager et al. 2013). LPC is then reacylated by the forward reaction of LPCAT. Because PC serves as a metabolic hub for further FA modification, C18:1 can be sequentially converted to C18:2 and C18:3. Accordingly, acyl editing plays a central role in PUFA production and the redistribution of modified FAs among different lipid pools. In metabolic engineering strategies for EPA and DHA production in plants, LPCAT has been used to mitigate the bottleneck caused by substrate dichotomy of fatty acid desaturases (FADs) and elongases. This intervention increases the proportion of omega 3-FAs in newly synthesized lipid pools and enhances the yield of VLC-PUFAs (Klińska-Bąchor et al. 2024a, b).

PC also serves as a precursor of DAG for TAG biosynthesis through the production of PC-derived DAG (Fig. 1a). Several mechanisms contribute to the formation of PC-derived DAGs, including head-group exchange between PC and DAG mediated by phosphatidylcholine: diacylglycerol cholinephosphotransferase (PDCT), as well as PC synthesis from de novo DAG and CDP-choline via choline phosphotransferase (CPT), followed by PC turnover through the reverse action of CPT or through phospholipase C (PLC)-mediated hydrolysis (Bates and Shockey 2025; Magdalena et al. 2021). In addition, FAs in PC can be further modified, transferred back to the acyl-CoA pool through acyl editing, or directly incorporated into TAG by PDAT (Bates and Shockey 2025).

In plant seeds, TAGs are stored in specialized organelles termed oil bodies, which consist of a hydrophobic TAG core enclosed by a phospholipid monolayer containing structural proteins such as oleosins (OLEs), caleosins and steroleosins. These proteins stabilize oil bodies and prevent their coalescence during seed desiccation and storage (Wu et al. 2010). Upon germination, TAGs are hydrolyzed by TAG lipases (TGLs), releasing FFAs and DAGs. The FFAs are subsequently converted to acyl-CoA esters and transported into the peroxisome via peroxisomal ABC transporter 1 (PXA1) (Fan et al. 2017; Wang et al. 2026a). Within the peroxisome, acyl-CoAs undergo successive cycles of β-oxidation to generate acetyl-CoA. This acetyl-CoA then enters either the glyoxylate cycle, thereby supporting gluconeogenesis and sugar synthesis, or the TCA cycle to drive energy production. Collectively, these processes supply the energy and carbon skeletons required for early seedling establishment and growth (Li et al. 2016; Park et al. 2023).

### Key enzymes in fatty acid and triacylglycerol biosynthesis

Extensive research in plants has identified key enzymes and regulatory components within lipid biosynthetic pathways that exert strong control over lipid accumulation. These include rate-limiting enzymes involved in de novo FA synthesis; desaturases that determine FA unsaturation, and acyltransferases that direct carbon flux into TAG assembly, as illustrated in Fig. 1a (Sagun et al. 2023; Zhou et al. 2024; Liu et al. 2024a, b).

#### Acetyl-CoA carboxylase in fatty acid biosynthesis

ACC functions as a key rate-limiting enzyme in the de novo FA biosynthetic pathway. It catalyzes the first committed step, namely the ATP-dependent carboxylation of acetyl-CoA to malonyl-CoA, thereby providing two-carbon units required for chain elongation by FA synthase (FAS) complex (Park et al. 2023). Functional studies have demonstrated its critical regulatory role in lipid accumulation.

#### Fatty acid desaturases: FAD2 and FAD3

Most newly synthesized C18:1-CoAs are first incorporated into PC and then further desaturated by ER-localized FADs. In plants, FAD2 (Δ12-desaturase) predominantly introduces a double bond at the Δ12 position of PC-bound 18:1 to produce PC-bound 18:2. Subsequently, FAD3 (omega-3/Δ15-desaturase) converts PC-bound 18:2 into PC-bound 18:3, which can be released to form 18:3-CoA (Park et al. 2023). The ER-localized production of FAs on PC represents the main route supplying PUFAs for TAG biosynthesis (Bates and Shockey 2025). Accordingly, most oilseed crops accumulate high levels of C18:2 PUFAs, particularly 18:2 and 18:3 (Magdalena et al. 2021).

Genetic manipulation of *FAD* genes has demonstrated their central role in determining FA composition. For example, RNA interference (RNAi) suppression of *OsFAD2-1* in rice resulting in an increase in C18:1 content and reductions in C18:2 and C16:0 levels in T_3_ grains (Zaplin et al. 2013). Conversely, overexpression of *FAD3* from rice and soybean under the control of the *GluC* promoter in rice increased ALA (C18:3) to 37.5% of total lipids (Liu et al. 2012; Tiwari et al. 2016). Collectively, these studies demonstrate that precise manipulation of *FAD2* and *FAD3* expression provides an effective molecular strategy for tailoring FA composition in major crops toward enhanced nutritional quality.

#### TAG synthetases: DGAT and PDAT

DGAT and PDAT catalyze the final step of TAG synthesis by converting DAG into TAG (Izadi-Darbandi et al. 2020; Cai et al. 2025). DGAT is widely regarded as a principal regulatory checkpoint controlling carbon flux toward TAG accumulation. Overexpression of DGAT1 from *Vernonia*, soybean or castor bean in Arabidopsis resulted in significantly higher seed oil content, underscoring its central role in enhancing lipid storage (Hatanaka et al. 2022). In contrast, accumulating evidence suggests that PDAT may exert a greater influence on oil composition than on total oil content. For instance, *PDAT* overexpression in alfalfa increased the proportions of linoleic and α-linolenic acids without significantly altering total lipid content (Marmon et al. 2017; Cai et al. 2025).

### Regulators of seed lipid biosynthesis: WRI1 and LEC1

Transcription factors that coordinate upstream carbon allocation and regulate the expression of lipid biosynthetic genes play central roles in determining lipid accumulation in plants. In addition, and structural proteins that enhance TAG storage efficiency and stabilize oil bodies further influence overall lipid content in plant. Together, these regulatory and structural components act synergistically to modulate both lipid biosynthesis and storage in plant tissues (Park et al. 2023).

#### Oil body proteins and TAG accumulation

Oil bodies are specialized organelles responsible for TAG accumulation in seeds. They are characterized by a phospholipid monolayer embedded with structural proteins, predominantly oleosins, on their surface. Oleosins play essential roles in maintaining oil body stability, preventing coalescence, and regulating lipid storage efficiency (Sadre 2024). Increasing evidence suggests that engineering oleosins can significantly increase oil content in plant biomass. Alterations in oleosin expression levels or protein structure can affect oil body formation and stability, thereby influencing TAG accumulation (Sadre 2024). For instance, embryo-specific expression of two soybean oleosin genes in transgenic rice led to the formation of more numerous and smaller oil bodies than in the wild type, resulting in increases in seed lipid content of up to 36.93% and 46.06% relative to the control, respectively (Liu et al. 2013). In contrast, RNAi-mediated suppression of two rice seed oleosin isoforms, *OLE16* and *OLE18,* caused the formation of large and irregular oil clusters and reduced TAG content (Wu et al. 2010).

#### Transcription factors of seed oil biosynthesis: WRI1 and LEC1

Transcription factors act as master regulators of diverse physiological and biochemical processes in plants. In the context of plant lipid biosynthesis, the most extensively studied transcription factors are *WRINKLED1 (WRI1)* and *LEAFY COTYLEDON1 (LEC1)* (Barthole et al. 2012). *WRI1* is a member of AP2/EREBP family and functions as a key regulator that activates genes involved in both glycolysis and de novo FA synthesis. Through this mechanism, WRI1 promotes carbon flux into lipid metabolism and plays a conserved role in enhancing plant seed oil accumulation (Barthole et al. 2012). *LEC1*, a pivotal member of the NF-YB family, regulates the accumulation of seed storage reserves by controlling the expression of genes associated with FA biosynthesis (Baud et al. 2009; Wei et al. 2025). Similarly, transgenic rice lines expressing coconut *WRI1* showed significantly increased seed oil content, accompanied by marked increases in C16:0 and C18:3 and a reduction in C18:1 (Sun et al. 2017). In maize, embryo-preferred expression of *ZmWRI1* increased seed oil content by 30.6% without compromising vegetative growth (Shen et al. 2010). By comparison, *ZmLEC1* overexpression increased oil content by 48.7% but also caused defects in germination and plant development, supporting a functional hierarchy in which *WRI1* acts downstream of *LEC1* (Shen et al. 2010). These findings highlight the strong potential of transcription factor-based engineering for improving lipid accumulation in cereal seeds. However, further studies are still needed to clarify the regulatory roles of transcription factors in determining cereal lipid composition. Coordinated engineering of *WRI1* and *LEC1* may therefore provide a promising system-level strategy for reprogramming carbon allocation and enhancing lipid production in cereals.

### Metabolic engineering for lipid biosynthesis in cereals

Cereals, particularly rice, maize and wheat, provide staple foods for a large proportion of the global population. However, their grains are inherently low in lipid content. Recent advances in genetic metabolic engineering have created new opportunities to enhance both lipid accumulation and FA quality in cereals (Barthole et al. 2012; Alameldin et al. 2017a; Liu et al. 2024a, b; Cui et al. 2025). Table 1 summarizes reported improvements in lipid content and composition achieved in cereals through metabolic and genetic interventions.

**Table 1:** Lipid engineering strategies and associated phenotypic effects in major cereals

Crop	Lipid trait altered	Tissue	Genes and edit type	Subcellular localization	Growth/phenotypic effects	References
**Rice**	↑TFA, ~ sixfold increase	Seed	*OsAGPL2*; RNAi	Cytoplasm	↓Yield; ↓starch; ↑lipid	(Wei et al. 2017)
	↓18:1, ↑16:0, 18:3	Seed	*CoWRI1*; OE	N.A	Normal growth; ↑oil and starch; ↓protein	(Sun et al. 2017)
	↑TFA, from 2.6% to 4.3%	Seed	*OsOle1*; OE	Lipid droplet; membrane	↓Biomass; flowering delayed, plants dwarfed; ↑lipid	(Bhattacharya et al. 2025)
	↑18:1, up to 41%; ↑16:0, up to 14%; ↓18:2, down to 41%	Seed	*AtDGAT1/AtWRI1/AtPDAT/AtOle*; OE	ER	Normal growth; ↑lipid	(Izadi-Darbandi et al. 2020)
	↑TFA, from 2.3% to 11.7%	Seed	*AtDGAT1/AGPL2*; OE	ER; plastid; mitochondria	↓Grain size; ↓1000-grain weight; ↓seed setting rate; ↑oil	(Liu et al. 2024a, b)
	↑TFA, from 19.5% to 46.1%	Seed	*GmOLE-A/GmOLE-B*; OE	ER; oil body	↓Biomass; ↑oil	(Liu et al. 2013)
	↑ALA, up to 6.4 mg/g	Seed	*OsFAD3*; OE	N.A	Normal growth;	(Cui et al. 2025)
	↑TFA, increase about 10%	Seed	*OsACBP2*; OE	Cytoplasm	Normal growth;	(Guo et al. 2019)
	↓Total wax, decrease 19%; ↓TFA, decrease 24%	Leaf	*OsPLS4*; point mutation	Chloroplast	↓Barrier function; ↑water loss; ↑leaf water permeability; ↑stress susceptibility; ↓total wax	(Zhou et al. 2020)
	↑TFA, increase 40%; ↑total membrane lipid, increase 20%	Leaf	*AtSFD1/GLY1*; OE	Plastid	Photosynthetic rate unchanged; ↑leaf TFA	(Singh et al. 2016)
	↓C16:0, C18:0, C18:1, C18:2, C18:3	Seed	*OsLTPL36*; RNAi	N.A	Embryo development delayed; ↓seed quality; ↓lipid content	(Wang et al. 2015)
	↑ALA, up to 46.4%; ↓18:2	Endosperm	*OsFAD3; GmFAD3-1*; OE	ER	↑ALA	(Liu et al. 2012)
	↑Total lipid, increase 27%	Seed	*OsmtSSB1*; point mutation	Mitochondria	↓Biomass; ↑protein, lipid, vitamins	(Li et al. 2021)
	↑ALA, 18:1; ↓18:2	Endosperm	*OsFAD3-1*; OE	ER	↑ALA	(Zhang et al. 2022)
	↑18:1, up to 80%; ↓18:2, down to undetectable	Brown rice	*OsFAD2-1*; KO	ER	Normal growth; ↑oleic acid, ↓linoleic acid	(Abe et al. 2018)
	↑Total lipid, up to 2.9%; ↑18:1, up to 55.0%; ↑18:2, up to 19.8%; ↑16:0, up to 16.8%	Endosperm	*OsFAD2-1*; RNAi	ER	Potential impact on seed development; ↓oil body-related proteins	(Tiwari et al. 2016)
	↑18:1; ↓18:2; ↓16:0	Seed	*OsFAD2-1*; RNAi	ER	Normal growth; ↑18:1, ↓18:2, ↓16:0	(Wu et al. 2025)
	↑18:1, from 51% to 65%; ↑18:2; from 12% to 30%; ↑16:0; from 14% to 17%; ↓14:0, down to 0.6%	Seed	*OsFAD2-1*; RNAi	ER	Normal growth; ↑18:1, ↓18:2, 16:0 and 14:0, ↑rice bran oil	(Zaplin et al. 2013)
**Maize**	↑EPA, up to 1.99%	Leaf	*Δ9-Elo*/*Δ8-Des*/*Δ5-Des*; OE	ER; cytoplasm	Impact on yield and grain quality not assessed; ↑EPA	(Wang et al. 2017)
	↑TFA, increase 71%; ↑total oil, increase 79%;	Leaf	*AtDGAT1*/*AtWRI1*/*AtOLE*; OE	ER; cytoplasm	Normal growth; ↑oil bodies	(Alameldin et al. 2017a)
	↑Total oil, up to 4.5%; ↑TAG, up to 23.7 mg/g; ↑18:1, increase 73.3%; ↓18:2, decrease 51.1%	Seed	*AtDGAT1/AtWRI1/AtOLE*; OE	ER; cytoplasm	Normal growth; ↓ω6/ω3 FA; ↓PUFA/SFA	(Alameldin et al. 2017b)
	↓16:0; ↓SFA; ↑UFA	Seed	*Zmfab*; mutant	N.A	Normal growth; total oil unchanged but SFA/UFA ratio altered	(Li et al. 2011)
	↑Total oil, from 35% to 48.7%;	Seed	*ZmLEC1*; OE	Nucleus	↓Germination and dwarfism; ↑oil	(Shen et al. 2010)
	↑total oil from 30.6% to 46%		*ZmWRI1*; OE		Normal growth; ↑oil	
	↑Seed oil, increase 17.1%; ↑embryo oil, increase 18.7%; ↑18:1, increase 61.3%; ↓18:2, decrease 24.1%	Seed	*DGAT1-2*; OE	ER	Normal growth; ↑Seed and endosperm oil	(Zheng et al. 2008)
	↑Total oil, increase 51.6%; ↑18:1, increase 33.2%; ↓16:0, decrease 30.2%	Seed	*DGAT1-2/FatB*; OE	ER; plastid	Normal growth; ↑high oil and FA composition	(Katral et al. 2024)
**Wheat**	↑TAG, from 0.7% to 6.4%; ↑18:1; ↓18:2	Endosperm	*AsWRI1*; OE	Nucleus	↓Biomass; ↑oil, ↓starch	(Snell et al. 2022)
	↓TFA, down to 20% of WT	Seed	*TaWR1L2*; RNAi	Nucleus	↓1000-grain weight; ↓TFA	(Yang et al. 2022)
	↑TAG, up to 30 mg/g	Endosperm	*ZmWRI1a*/*AtDGAT1*/*SiOLE*; OE	Nucleus; ER	Normal growth; ↑TAG	(Larkin et al. 2021)
	↑ TAG, ~ ninefold increase;	Endosperm	*AsWRI1*; OE	Nucleus; ER	Abnormal grain development; ↑oil, ↓starch, ↓sugar	(Grimberg et al. 2020)
	GLA, 0.32% of TFA	Seed	Synthetic Δ6-des *D6D*; OE	ER	Normal growth; GLA and SDA successfully synthesized	(Mihálik et al. 2015)
	The highest GLA	Awn				
	The highest SDA	Root				

This table summarizes lipid engineering researches in major cereals

↑: increase; ↓: decrease, *OE* overexpression, *N.A.* unknown, *TFA* total fatty acid

#### Lipid composition and distribution in cereal seeds

In most plant seeds, FAs and TAGs are composed predominantly of C_16_ and C_18_ acyl chains. Cereal grains contain two major zygotic tissues, the embryo and the endosperm. The endosperm functions primarily as a storage tissue for starch and proteins, whereas lipids are stored mainly in the embryo in the form of TAGs (Barthole et al. 2012; Zhang et al. 2022). Among major cereals, the total lipid content of rice (*Oryza sativa*), wheat (*Triticum aestivum*) and barley (*Hordeum vulgare*) is typically 2%–3% of seed dry weight, whereas maize generally contains a higher level, at approximately 4%. The FA profiles of these cereals are dominated by C16:0, C18:1and C18:2. In maize, wheat, and barley, these FAs account for approximately 10%–20%, 15%–30% and 50%–60% of total FAs, respectively, whereas in rice they accounted for about 20%, 30%–40% and 30%–40%, respectively (Barthole et al. 2012; Ohlrogge et al. 2018; Chang et al. 2024).

#### Strategies for increasing lipid accumulation in cereals

Because cereals possess only a limited natural capacity for lipid accumulation, metabolic engineering strategies have been developed to overcome these constraints. Based on the compartmentalized nature of plant lipid metabolism, the integrated “push–pull-package-protect” framework has emerged as a widely adopted strategy for increasing TAG content and modify FA composition (Sagun et al. 2023). In this framework, the push component enhances de novo FA synthesis in the plastid; pull promotes assembly of FFs into TAG in the ER; package facilitates the sequestration of TAG into stable cytoplasmic oil bodies; and protect preserve oil body integrity and limits TAG turnover (Fig. 1b) (Vanhercke et al. 2019; Sagun et al. 2023; Zhou et al. 2024). In practice, these strategies are often combined to maximize both oil accumulation and composition improvement. For example, in rice, a combined push–pull-package-protect approach was implemented through constitutive overexpression of *AtWRI1, AtDGAT1*, *AtPDAT* and *AtOle.* In this system, *AtWRI1*, stimulated FA synthesis, *AtDGAT1* and *AtPDAT* promoted the final step of TAG assembly, and *AtOle* enhanced oil body formation and TAG protection. This multigene strategy increased seed TAG content by 26% relative to the control, while C18:1 and C16:0 levels rose by 28% and 27%, respectively. Overall, total oil content increased by 70% in seeds and 22.5% in leaves (Izadi-Darbandi et al. 2020). These findings illustrate the value of rationally combining complementary engineering strategies to target specific lipid traits.

#### Genetic transformation and genome editing toolkits for lipid engineering in major cereals

Efficient transformation and genome-editing systems are essential for metabolic reprogramming in major cereals. The heterologous production of high-value lipids that are not naturally abundant in cereals requires not only the introduction of exogenous biosynthetic pathways but also precise manipulation of endogenous lipid metabolism. In rice, maize, and wheat, *Agrobacterium*-mediated transformation and particle bombardment remain the principal methods for stable transgene integration, whereas CRISPR-Cas-based genome editing has become an increasingly important tool for redirecting metabolic flux, eliminating competing pathways, and fine-tuning gene expression (Chen et al. 2014). Together, these technologies enable multigene stacking, tissue-specific expression, and coordinated regulation of lipid biosynthetic modules, thereby supporting rational engineering of both lipid quantity and composition in cereals.

Among cereal species, rice has been the most amenable to transgenic and genome-editing approaches for lipid engineering, owning in part to its relatively high transformation efficiency and smaller genome. In rice, these tools have been used to enhance TAG accumulation and modify FA composition through coordinated manipulation of FA biosynthesis, desaturation, and TAG assembly pathways. In addition, the use of strong or seed-preferential promoters can improve transgene expression during grain development and thereby enhance metabolic output (Abe et al. 2018; Guo et al. 2019; Izadi-Darbandi et al. 2020; Liu et al. 2024a, b). In maize, transformation strategies have been widely applied to genes encoding desaturases, elongases, and acyltransferases in order to alter oil composition, improve nutritional quality, and investigate lipid functions during reproductive development. Particle bombardment is still frequently used in maize, partly because of the technical difficulties associated with *Agrobacterium* transformation of this species. However, the efficiency and stability of multigene engineering can be constrained by the size and complexity of the introduced expression cassette, making vector design a critical factor in successful metabolic engineering., The use of tissue-specific promoters, including leaf-preferential promoters, can further improve spatial and developmental control of transgene expression in maize (Shen et al. 2010; Alameldin et al. 2017b). Compared with rice and maize, wheat remains more recalcitrant to transformation, which has limited the pace of metabolic engineering in this crop. Nevertheless, CRISPR-Cas9 has emerged as a promising platform for targeted genome modification in wheat. In parallel, inducible promoters provide opportunities for conditional metabolic regulation under specific developmental or environmental contexts, whereas RNA interference offers an additional approach for fine-tuning endogenous gene expression and improving oil quality traits (Grimberg et al. 2020; Larkin et al. 2021; Snell et al. 2022; Yang et al. 2022).

Collectively, these advances indicate that major cereals possess sufficient metabolic plasticity to support lipid pathway reprogramming and can therefore be developed as chassis for the heterologous production of high-value compounds such as omega-3 polyunsaturated FAs, WEs, and pheromones. Continued progress will depend on the selection of appropriate transformation and editing platforms, the optimization of promoter systems, and improved strategies for stable multigene expression. These factors will be central to the development of efficient cereal-based platforms for high-value lipid production.

#### Progress in engineering lipid biosynthesis in cereals

Recent progress in cereal lipid engineering has focused on three major objectives: increasing total oil content, optimizing FA composition, and introducing heterologous pathways for the synthesis of nutritionally valuable FAs. Collectively, these efforts highlight the potential of cereals as platforms for producing both food and value-added lipid products.

Substantial increases in oil content have been achieved through multigene engineering approaches that redirect carbon flux from starch biosynthesis toward lipid production while optimizing intracellular lipid storage (Yang et al. 2019; Izadi-Darbandi et al. 2020; Liu et al. 2024a, b; Wang et al. 2026b). In rice, endosperm-specific overexpression of *AtDGAT1* under the *Glb1* promoter, combined with CRISPR-Cas9-mediated knockout of *AGPL2,* a rate-limiting gene in starch biosynthesis, and *MTSSB1,* a regulator of aleurone layer thickness, increased grain oil content by more than fivefold, from 2.33% to 11.72% of dry weight (Liu et al. 2024a, b). This result demonstrates that substantial carbon reallocation toward lipids can be achieved without major penalties to agronomic performance. In maize, constitutive co-expression of *AtDGAT1*, *AtWRI1*, and *AtOleosin* increased seed TAG content by 117% and total leaf oil content by 25%, whereas embryo-specific expression of *ZmWRI1* increased seed oil content by 30.6% without adversely affecting vegetative growth, underscoring the importance of tissue-specific regulation (Shen et al. 2010; Alameldin et al. 2017a). In *Sorghum bicolor,* constitutive expression of *UrDGAT2a*, and sesame *Oleosin-L*, together with leaf mesophyll-specific expression of *ZmWRI1*, increased TAG accumulation in vegetative tissues to 3%–8.4% of dry weight cross different developmental stages. The best performing line reached 6.9% total FAs and 4.6% TAG at the boot leaf stage. This approach supports the concept of dual-use crops capable of producing both grain and lipid-rich biomass. However, these modifications may also undesirable phenotypes, including reduced transitory starch, dwarfism and impaired fertility, emphasizing the metabolic trade-offs associated with enhanced lipid accumulation. These limitations highlight the need for precise spatial and temporal control of transgene expression (Vanhercke et al. 2019).

In parallel with increasing total oil content, considerable effort has been directed toward improving FA composition for enhanced nutritional quality (Liu et al. 2012; Cui et al. 2025; Wu et al. 2025). In rice, endosperm-specific overexpression of the ER-localized genes *GmFAD3-1* and *OsFAD3-2* increased seed α-linolenic acid (ALA) content from 0.36 to 8.57 and 10.06 mg/g, respectively, levels that are sufficient to meet most daily dietary requirements (Liu et al. 2012). Notably, intragenic engineering using the endogenous rice *FAD3* gene driven by the endosperm-specific *GluC* promoter increased seed ALA content 14.93-fold, to 6.44 mg/g, without introducing foreign DNA, thereby potentially reducing biosafety and regulatory concerns associated with transgenic approaches (Cui et al. 2025).

Another important objective has been the heterologous production of VLC-PUFAs, which are valuable for human nutrition and sustainable aquaculture but remain challenging to produce efficiently in plants (Cheng et al. 2010a, b; Yang et al. 2025). In maize, the introduction of the alternative Δ8 desaturation pathway enabled the biosynthesis of EPA, which is absent from wild type plants (Wang et al. 2017).This work demonstrates the potential of cereals as sustainable platforms for the production of nutritionally important VLC-PUFAs.

Overall, these studies show that metabolic engineering can substantially enhance both the quantity and quality of lipids in cereals. Future progress will likely depend on increasingly precise strategies that coordinate carbon partitioning, lipid assembly, and storage while minimizing negative effects on plant growth and yield.

## Heterologous production of high-value lipid-derived compounds in plants

Reconstruction of heterologous metabolic pathways in plants is an important strategy for the large-scale production of high-value lipid-derived compounds. Building on the transformation and genome-editing toolkits described above, plants can be engineered not only to enhance endogenous lipid accumulation but also to synthesize entirely new classes of metabolites. Among the most intensively studied targets are omega-3 PUFAs, WEs and insect sex pheromones, owing their broad applications in human nutrition and health, industrial lubricants and surfactants, and environmentally sustainable pest management. Table 2 summarizes representative examples of the heterologous production of EPA, DHA, WEs, and insect sex pheromones, together with their major biosynthetic intermediates, in plant systems. Table 3 compares cereals (rice, wheat, maize) with established oilseed platforms (*Camelina*, *Brassica*, *Crambe*) across five key parameters. Transformation efficiency is highly species- and protocol-dependent, with rice showing particularly efficient transformation systems among cereals, whereas several oilseed platforms provide well-established seed-oil engineering chassis. Oilseed platforms generally have substantially higher endogenous seed oil content and stronger carbon allocation toward lipid accumulation. By contrast, cereals have lower seed oil content (approximately 2.2%–4% DW) and starch-dominated carbon partitioning, but their large vegetative biomass and sugar-rich tissues may provide advantages for scalable non-seed lipid production. Regulatory requirements also differ substantially across jurisdictions, particularly among China, the USA, and the EU.

**Table 2:** Summary of cases of heterologous production of EPA/DHA, wax esters and sex pheromones in plants

Substances	Plant platforms and tissues	Lipid content modification and targets production	Transformed gene(s)	References
**DHA/EPA**	*C. sativa*; seed	DHA: up to 12.4% of TFA	Δ6-FAD (*Micpu-*Δ6D)/Δ6-ELO (*Pyrco-*Δ6E)/ Δ5-ELO (*Pyrco-*Δ5E)/ Δ5-FAD (*Pavsa-*Δ5D)/Δ4-FAD (*Pavsa-*Δ4D)	(Petrie et al. 2014)
	*C. sativa*; seed	ALA: increase 50%	*PfFAD3-1*	(Park et al. 2023)
	*C. sativa*, seed	EPA: max 31% of TFA	Δ6-FAD (*OtA6*)/Δ6-ELO (*PSE1*)/Δ5-FAD (*TcΔ5*)/ Δ12-FAD (*PsΔ12*)/omega-3 FAD (*Piω3*)	(Ruiz‐Lopez et al. 2014)
		EPA: max 12% of TFA DHA: max 14% of TFA	Δ6-FAD (*OtA6*)/Δ6-ELO (*PSE1*)/Δ5-FAD (*TcΔ5*)/ Δ12-FAD (*PsΔ12*)/ omega-3 FAD (*Piω3*)/Δ5-ELO (*OtElo5*)/Δ4-FAD (*EhΔ4*)	
	*C. sativa*, seed	EPA: about 9% of TFA DHA: 3.5% to 6.5% of TFA EPA + DPA + DHA > 20% TFA	Δ6-FAD (*OtA6*)/Δ6-ELO (*PSE1*)/Δ5-FAD (*TcΔ5*)/ Δ5-ELO (*OtElo5*)/ Δ4-FAD (*RCC809*)/Δ12-FAD (*PsΔ12*)/ omega-3 FAD (*Piω3*)	(Han et al. 2020)
	*C. sativa*, seed	DHA and EPA DPA: up to 0.8% of TFA	Δ15-FAD (*PerfΔ15*)	(Han et al. 2022)
	*C. sativa*, seed	Total omega-3 FA: up to 39.2% of TFA EPA: up to 16% of TFA ALA: down to 17.7% of TFA LA: down to 11.4% of TFA	Δ6-FAD (*OtA6*)/Δ6-ELO (*PSE1*)/ Δ5-FAD (*TcΔ5*)/omega-3 FAD (*Hpω3*)	(Usher et al. 2017)
		Total omega-3 FA: up to 38.8% of TFA EPA + DHA + DPA up to 15% of TFA 18:1 down to 7% of TFA, 18:2 increase to 21% of TFA	Δ6-FAD (*OtA6*)/Δ6-ELO (*PSE1*)/Δ5-FAD (*TcΔ5*)/ omega-3 FAD (*Hpω3*)/Δ5-ELO (*OtElo5*)/Δ4-FAD (*EhΔ4*) Δ12-FAD (*PsΔ12*)	
	*C. sativa*, seed	EPA: up to 14.8% of TFA (single plant)	Δ9-ELO (*EhElo9*)/Δ9-ELO (*IgElo9*)*/*Δ8-FAD (*PsA8*)/ Δ5-FAD (*EhΔ5*)/Δ12-FAD (*PsΔ12*)/omega-3 FAD (*Piω3*)	(Ruiz-Lopez et al. 2015)
	*N. benthamiana*, leaf	EPA: up to 1.4% of total lipid	Δ6-FAD (*OtDES6*)/Δ6-ELO (*PSE*)/ Δ5-FAD (*PtDES5*)/*PtLPCAT*	(Klińska-Bąchor et al. 2024a)
	*N. benthamiana*, leaf	EPA: up to 9.2% of TFA ETA: up to 2.1% of TFA	*PtDGAT2b*/Δ6-FAD (*OtD6*)/Δ6-ELO (*PSE*)/ Δ5-FAD (*TcD5*)	(Klińska-Bąchor et al. 2024b)
		EPA: up to 7.9% of TFA ETA: up to 4.0% of TFA	*PtDGAT2b*/Δ6-FAD (*OtD6*)/Δ6-ELO (*PSE*)/Δ5-FAD (*PtD5*)	
	*A. thaliana*, seed	DHA: up to 15.1% of TFA	Δ12-FAD (*Lackl-*Δ12D)/Δ6-FAD (*Micpu-*Δ6D)/ omega-3 FAD (*Picpa-*ω3D)/Δ6-ELO (*Pyrco-*Δ6E)/ Δ5-ELO (*Pyrco-*Δ5E)/Δ5-FAD (*Pavsa-*Δ5D)/ Δ4-FAD (*Pavsa-*Δ4D)	(Petrie et al. 2012)
	*B. napus*, seed	DHA: up to 3.87% EPA: up to 0.79%	*PFA1*/*PFA2*/*PFA3*/*NoHet1*	(Walsh et al. 2016)
	*A. thaliana*, seed	DHA up to 1.7% of TFA	*PFA1*/*PFA2*/*PFA3*/*NoHet1*	
	*G. max*, seed	DHA: up to 2.7% of TFA EPA: up to 1.5% of TFA	*PFA1*/*PFA2*/*PFA3*/*NoHet1*	
**Wax esters**	*A. thaliana*, seed	WT: Wax ester production increased significantly	*MmFAR1*/*MmFAR1Δc*/*MmWS*/*Oleo3*	(Heilmann et al. 2012)
		*fae1 fad2* mutant: olely-oleate > 65% of wax esters	*MmFAR1*/*MmFAR1Δc*/*MmWS*/*Oleo3*	
	*A. thaliana*, seed;	23 mg/g	*ScWS-MaFAR*	(Yu et al. 2018)
		35 mg/g	*ScWS-MaFAR/ScWS-MaFAR*	
		64 mg/g	*ScWS-MaFAR/MaFAR*	
		4 mg/g	*MaFAR/AbWSD1*	
		12 mg/g	*MaFAR/PCOAbWSD1*	
		17 mg/g	*MaFAR/TMMmAWAT2-AbWSD1*	
		14 mg/g	*MaFAR-MaWS2*	
	*C. sativa*, seed	> 40 mg/g	*ScWS-MaFAR*	
	*A. thaliana*, seed	WT: 108 mg/g	*MaFAR/ScWS*	(Iven et al. 2016)
		*fae1 fad2* mutant: 86 mg/g	*MaFAR/ScWS*	
	*C. sativa*, seed	47 mg/g	*MaFAR/ScWS*	
	*C. sativa*, seed	MoMa10: 74.6 nmol/seed	*MaFAR*/*MmWS*/*Thio10*	(Ruiz-Lopez et al. 2017)
		MoMa14: 77.6 nmol/seed	*MaFAR*/*MmWS*/*Thio14*	
	*C. abyssinica*, seed	up to 30% of total lipid	*ScFAR/ScWS*	(Zhu et al. 2016)
	*B. carinata*, seed	C22 and C24 monounsaturated acids/oils: increased by 2 times C20 monounsaturated acid: reduced about 50%	*ScFAR*/*ScWS*/*LaFAE1*	
	*C. sativa*, seed	up to 30% of total lipid	*ScFAR*/*ScWS*	
	*C. abyssinica*, seed	18%–40% total oil; single seed 12.4%–39.7%	*ScFAR*/*ScWS*	(Li et al. 2019)
		5.8%–30% total oil; seed yield decreased	*ScFAR*/*ScWS*/*ScFAE1*	
		23.1%–33.2% total oil; single seed 15.7%–38.8%	*ScFAR*/*ScWS*/*ScFAE1*/*CaFAD2*	
	*B. carinata*, seed	up to 25.6% total neutral lipids	*ScFAR*/*ScWS*	(Tesfaye et al. 2024)
	*L. campestre*, seed	6.3–44.7 mg/g	*ScFAR*/*ScWS*	(Ivarson et al. 2017)
		20.1–85.8 mg/g	*ScFAR*/*ScWS*/*ScFAE1*	
	*N. benthamiana*, leaf	1.62 nmol/mg	*MaFAR*/*AtPES2*	(Aslan et al. 2014)
		0.66 nmol/mg	*MaFAR*/*MhWS*	
		0.5 nmol/mg	*AtFAR6/AtPES2*	
	*N. benthamiana*, leaf	0.2–0.4 µg/mg	*ScFAR*/*ScWS*	(Whitehead et al. 2025)
	*A. thaliana*, seed	2–3 µg/mg	*ScFAR*/*ScWS*	
		4–5 µg/mg	*ScFAR*/*ScWS*/*ScLDAP1*	
	*N. benthamiana*, leaf	~ sixfold increase	*MaFAR*/*MhMS*	(Aslan et al. 2015b)
	*N. benthamiana*, leaf	73% increase	*KASII*/*AtFAR6/AtPES2*	(Aslan et al. 2015a)
**Insect pheromones**	*N. benthamiana*, leaf	Z11-16:OAc: 1280 µg	*AtrΔ11*/*HarFAR*	(Ding et al. 2014)
	*N. benthamiana*, leaf	Product: 16:0, Z9,Z12,Z15-18:OH	*CsupFAR2*	(Xia et al. 2022a, b)
		Product: Z11-16:OH	*CsupYPAQ*/*CsupFAR2*	
		Product: Z9-16:OH	*CsupKPSE*/*CsupFAR2*	
		Product: Z11-16:OH, Z11-16:Ald	*CsupYPAQ*/*CsupFAR2*/*CsupKPSE*, *Csup15570*	
		Product: Z11-16:OH, Z11-16:Ald, Z11-16:OAc	*CpuFatB1*/ *AtrΔ11*/ *CsupYPAQ*/ *CsupFAR2*/*ATF1*	
	*N. benthamiana*, leaf	Z11-16:OH: 111.4 ± 13.7 μg/g of fresh leaf Z11-16:OAc: 11.8 ± 1.3 μg/g of fresh leaf	*AtrΔ11*/*HarFAR*/*EaDAct*	(Mateos-Fernández et al. 2021)
	*N. tabacum*, leaf	Z11-14:acid: 0.1%–0.4% of TFA	*CpaFatB2*/*AveΔ11*	(Xia et al. 2020)
		E11-14:acid: 0.1%–0.2% of TFA	*CpaFatB2*/*CpaE11*	
		Z11-16:acid: 0.1%–0.9% of TFA	*CpuFatB1*/*AtrΔ11*	
	*N. benthamiana*, leaf	Z11-16:acid: up to 17.6% of TFA	*CpuFatB1*/*AtrΔ11*	
	*C. sativa*, seed	Z11-16:1: 20% of TFA	*CpuFatB1*/*AtrΔ11*	(Wang et al. 2022)
	*C. sativa*, seed	E9-12:acid: 2.5% of TFA, E8,E10-12:acid: 0.22% of TFA;	*UcTE*/*CPRQ_Cpo*/*AtWRINKLED1*/ *CvLPAAT*	(Xia et al. 2021)
		E9-12:acid: 2.4% of TFA, E8,E10-12:acid: 0.22% of TFA;	*CPRQ_Ath*	
		E9-12:acid: 3.4% of TFA, E8,E10-12:acid: 0.31% of TFA;	*CPRQ_Ath*/*UcTE*/*P19*/*CPRQ_Cpo*	
		E9-12:acid: 9.4% of TFA, E8,E10-12:acid: 5.5% of TFA	*CPRQ_Ath*/*CPRQ_Osa*/*P19*/*CPRQ_Cpo*	
	*N. benthamiana*, leaf	Nepetalactone release: 17.4 ng/g FW/h	*GPPS*/*GES*	(Ontiveros-Cisneros et al. 2025)
		Nepetalactone release: 4.66 ng/g FW/h	*GPPS*/*GES*/*G80*/*8HGO*/*ISY*/*MLPL*	
	*C. sativa*, seed	Nepetalactone release: 1.62 ng/g FW/h	*GPPS*/*GES*	
		Nepetalactone release: 1.54 ng/g FW/h	*GPPS*/*GES*/*G80*/*8HGO*/*ISY*/*MLPL*	

This table summarizes cases of heterologous production of EPA/DHA, wax esters and sex pheromones in plant platforms. *TFA* total fatty acid, *ELO* elongase, *FAD* fatty acid desaturase, *FW* fresh weight. *A. thaliana*: *Arabidopsis thaliana*; *B. carinata*: *Brassica carinata*; *B. napus*: *Brassica napus*; *C. abyssinica*: *Crambe abyssinica*; *C. sativa*: *Camelina sativa*; *G. max*: *Glycine max*; *L. campestre*: *Lepidium campestre*; *N. benthamiana*: *Nicotiana benthamiana*; *N. tabacum*:* Nicotiana tabacum*

**Table 3:** Comparative features of cereals and established oilseed platforms for lipid metabolic engineering

	**Cereals (Rice/Wheat/Maize)**			**Oilseeds platforms (Camelina/Brassica/Crambe)**		
**Transformation efficiency**	Rice	Indica 69%–83%; Japonica ~97%	(Rengasamy et al. 2024)	Camelina	Floral dip: up to 4.12%,; Immature zygotic embryo: 13%–17%	(Van Belle 2020) (Rezaeva et al. 2025)
	Wheat	Efficiency range 1.5%–51%; Fielder up to 25%; Haploid embryo: 66.7%–78.4%; GRF4-GIF1: Fielder up to 62%–77.5%	(Zhang et al. 2025) (Hayta et al. 2019) (Han et al. 2025) (Debernardi et al. 2020) (Hayta et al. 2026)	Brassica	Agrobacterium-mediated transformation: 2.2%–54.2%	(Dai et al. 2020) (Liu et al. 2011) (Naeem et al. 2020) (Roy et al. 2026) (Cao et al. 2020)
					Floral spray: 10%–30% Particle bombardment (chloroplast transformation): ~ 0.3%	(Aminedi et al. 2019) (Cheng et al. 2010a, b)
	Maize	Ternary vector system + GRF-GIF chimera; 2.3%–47.4% CRISPR/Cas9: 11%–80%; Pmi-o optimized method: 9.37%–11.33%	(Vandeputte et al. 2024) (Liu et al. 2025b) (Wang et al. 2025)	Crambe	Agrobacterium-mediated transformation: 1.3%–10% Hairy root method 16%–45%	(Qi et al. 2014) (Głąb et al. 2013) (Chhikara et al. 2012) (Li et al. 2010) (Li et al. 2013) (Miklaszewska et al. 2017)
**Endogenous seed oil content (% DW)**	Rice	~ 3%	(Liu 2011)	Camelina	28%–50%	(Zanetti et al. 2021)
	Wheat	~ 2.2%	(Liu 2011)	Brassica	~ 45%	(Weselake et al. 2024)
	Maize	~ 4%	(Oakes et al. 2011)	Crambe	23.4%–36.6%	(Karpaviciene et al. 2020)
**Carbon partitioning (starch vs. oil)**		Strongly favors starch;	(Grimberg 2014)		Strongly favors oil;	(Shen et al. 2025)
**Regulatory considerations**	Rice	China: stepwise approval; GM rice non-commercialized; USA: Product-based regulation; GE crops exempted EU: stringent GMO regulation	(Liang et al. 2022) (Mou et al. 2025) (Eriksson et al., 2019) (Smyth 2020)	Camelina	China: no specific approval status indicated; USA: product-based; risk-oriented regulation; novel gene-edited oilseeds remain uncertain; EU: stringent GMO regulation	(Eriksson et al., 2019) (Smyth 2020) (Liang et al. 2022)
	Wheat	China: GE first approved for staple food in 2024; GM at production trial stage USA: product-based; less commercialized than maize; EU: stringent GMO regulation	(Liang et al. 2022) (Smyth 2020) (Eriksson et al., 2019) (Mou et al. 2025)	Brassica	China: no domestic commercialization cultivation approval; USA: product-based; risk-oriented regulation; EU: stringent GMO regulation	(Liang et al. 2022) (Eriksson et al., 2019) (Smyth 2020)
	Maize	China: stepwise approval; GM maize in large-scale cultivation USA: mature commercial GM maize under product-based regulation EU: stringent GMO regulation	(Liang et al. 2022) (Mou et al. 2025) (Smyth 2020) (Eriksson et al., 2019)	Crambe	China: no specific approval status indicated; USA: long history of oilseed cultivation, product-based risk-oriented regulation; novel gene-edited oilseeds remain uncertain; EU: stringent GMO regulation	(Liang et al. 2022) (Eriksson et al., 2019) (Smyth 2020)
**Suitability for non-seed tissues (leaf/stem oil)**	Highly favorable	High biomass; Established agricultural infrastructure; Suitable “biomass refineries”	(Alameldin et al. 2017a) (Mathew et al. 2017) (Izadi-Darbandi et al. 2020)	Limitation	Seed-centric metabolism; Intrinsically low oil accumulation in vegetative tissues	(Xu and Shanklin 2016) (Vanhercke et al. 2019)

*GM* Genetically modified (through recombinant DNA technology), *GE* Gene-edited (through targeted mutagenesis without introducing foreign DNA), *GMO* Genetically modified organism

### Production of EPA and DHA

EPA and DHA are VLC omega-3 PUFAs that play essential roles in human health (Metz et al. 2001). In nature, these FAs are synthesized primarily by aquatic microorganisms, including algae, bacteria, and protists, and subsequently accumulate in higher trophic-level organisms through the food chain (Qiu et al., 2001). In plant metabolic engineering, EPA biosynthesis is typically reconstructed through the ALA-derived omega-3 pathway. In this pathway, ALA is converted to stearidonic acid (SDA) by Δ6-desaturase, elongated to form eicosatetraenoic acid (ETA) by Δ6 elongase, and then desaturated by Δ5-desaturase to produce EPA (Abe et al. 2006). Although this pathway has been successfully introduced into plants, EPA accumulation is often constrained by limited heterologous enzyme activity and competition with endogenous FA metabolism (Wu et al. 2005; Usher et al. 2015). By comparison, DHA biosynthesis is more complex and does not occur naturally in terrestrial plants. In microorganisms and certain algae, DHA is generally produced from EPA via elongation to docosapentaenoic acid (DPA) followed by Δ4 desaturation (Qiu et al. 2001; Vaezi et al. 2013). In contrast, mammals produce DHA through a Δ4-desaturase-independent pathway involving elongation, Δ6 desaturation, and β-oxidation (Sprecher 2002). Because higher plants lack both the relevant Δ4-desaturase and associated elongation activities, DHA production in plant systems requires the introduction of heterologous genes to reconstruct a functional pathway (Petrie et al. 2012; Meesapyodsuk et al. 2018).

Increasing global demand for omega-3 PUFAs in human nutrition and aquaculture has stimulated the development of sustainable plant-based production systems (Metz et al. 2001). To date, the greatest progress has been achieved in the oilseed crops *Brassica napus* and *Camelina sativa*. Transgenic lines of these species have been developed that accumulate up to 19% EPA + DHA of total FAs, whereas DHA alone has reached approximately 12% in Camelina and combined EPA + DHA around 4% in canola (Petrie et al. 2014; Walsh et al. 2016). At present, plant-derived EPA and DHA are being developed primarily for aquafeed applications, although direct food uses are also under investigation (West et al. 2019). Plant-derived omega-3 LC-PUFA production has recently moved beyond proof-of-concept studies toward commercialization. Nuseed’s omega-3 canola event NS-B50027-4 represents the most advanced case, with Aquaterra® commercialized for aquafeed applications and Nutriterra® advancing for human nutrition through major regulatory milestones (Gao and Bai 2025). The omega-3 Camelina platform developed by Professor Napier Johnathan’s group has also progressed through commercial licensing and U.S. regulatory advancement, making it one of the closest camelina-based systems to commercialization (Napier et al. 2013). By contrast, the BASF/Cargill LBFLFK omega-3 canola event has obtained regulatory clearance in the United States but has not yet reached broad commercial deployment (https://www.federalregister.gov/documents/2019/08/07/2019-16921/basf-plant-science-lp-determination-of-nonregulated-status-of-canola-genetically-engineered-for). These developments suggest that oilseed platforms are currently much closer to commercialization than cereal-based EPA/DHA production systems.

Rice and maize, as two of the world's major cereals, present similar metabolic constraints for EPA and DHA synthesis. Although both contain appreciable amounts of linoleic acid, they generally accumulate only low levels of ALA, typically less than 2%, thereby limiting precursor availability for omega-3 LC-PUFA biosynthesis. Moreover, like other terrestrial plants, they lack the endogenous desaturase and elongase systems required to convert ALA into EPA and DHA (Goffman et al. 2003; Qi et al. 2004; Ruiz-Lopez et al. 2015; Wang et al. 2017). Accordingly, engineering strategies in cereals have been focused on heterologous expression of key desaturases and elongases from algae, fungi and other marine organisms. In rice, an important intermediate objective has been to increase ALA content as a means of enhancing precursor supply for downstream LC-PUFA biosynthesis. Liu et al. (2012) introduced several omega-3 (Δ-15) *FAD* genes from rice and soybean into rice under the control of endosperm-specific or constitutive promoters. Overexpression of the ER-localized *GmFAD3-1* and *OsFAD3-2* genes increased seed ALA content from 0.36 to 8.57 and 10.06 mg/g, respectively, corresponding to 23.8-fold and 27.9-fold increases over the non-transformed control. More recently, Cui et al. (2025) used a double T-DNA strategy to express *OsFAD3* specially in the rice endosperm, generating ALA-enriched rice with a seed ALA content of 6.44 mg/g, approximately 15-fold higher than that of the wild type. Feeding experiments further showed that consumption of this rice increased ALA, EPA, and DHA levels in rat serum and brain tissue, highlighting the nutritional potential of plant-based omega-3 biofortification (Cui et al. 2025). In maize, metabolic engineering has focused more directly on reconstructing heterologous EPA biosynthesis. Using particle bombardment, Wang et al. introduced genes encoding Δ9-elongase from *Isochrysis galbana*, Δ8-desaturase from *Euglena gracilis*, and Δ5-desaturase from *Mortierella alpina*, thereby enabling EPA biosynthesis through an alternative Δ8-pathway in leaves. In the engineered plants, EPA reached 1.99% of total FAs (Wang et al. 2017). Although seed EPA accumulation was not examined, this study demonstrated the feasibility of heterologous EPA production in maize and established a basis for future grain-targeted engineering. More broadly, successful production of omega-3 LC-PUFAs in cereals will likely require not only multigene stacking of heterologous desaturases and elongases but also genome-editing strategies, including CRISPR-Cas9-mediated suppression of endogenous competing pathways, to improve precursor allocation and metabolic flux toward EPA and DHA biosynthesis (Haslam et al. 2016; Troncoso-Ponce et al. 2016).

Overall, these studies indicate that cereals can serve as prospective hosts for omega-3 LC-PUFA engineering, although significant metabolic and regulatory constraints remain. Further progress will depend on improving precursor supply, coordinating multigene pathway expression, and optimizing tissue-specific accumulation.

### Production of wax esters

**Fig. 2:**
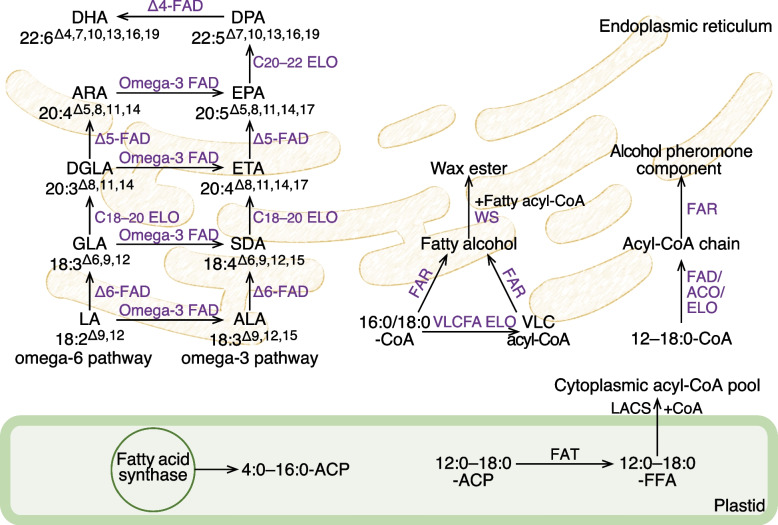
Metabolic engineering synthesis routes of PUFAs (EPA and DHA), wax esters, and type I sex pheromones in plants. Following synthesis in the plastid, FFAs are transported to cytoplasm and activated to acyl-CoA. The cytoplasmic acyl-CoA pool serves as substrates for heterologous lipid synthesis. PUFAs: the endogenous C18:2 is further desaturated by desaturases. C_18–20_ and C_20–22_ elongases specific to Δ6- and Δ5-desaturated fatty acyl. Wax esters: C_16_ or C_18_ acyl-CoA are elongated to VLC acyl-CoA via elongases. These acyl-CoA chains are subsequently reduced to fatty alcohols by reductases. Esterification of fatty alcohols with fatty acyl chains catalyzed by WS to synthesize WEs. Type I sex pheromones: via desaturases, elongases and β-oxidation enzymes, acyl-CoAs are catalyzed to form acyl-CoA chain pheromone intermediates with various carbon length and desaturation, FARs catalyze the latter to produce alcohols, which could be subjected to further modification to produce corresponding acetate or aldehyde pheromone compounds by acetyltransferase or alcohol oxidase/dehydrogenase. Abbreviation: ACP: the acyl carrier protein; ACO: acyl-CoA oxidase; ALA: α-linolenic acid; ARA: arachidonic acid; DHA: docosahexaenoic acid; DGLA: dihomo-γ-linolenic acid; DPA: docosapentaenoic acid; ELO: fatty acid elongase; EPA: eicosapentaenoic acid; ETA: eicosatetraenoic acid; FAD: fatty acid desaturase; FAR: fatty acid reductase; FAT: fatty acyl-ACP thioesterase; FFA: free fatty acid; GLA: γ-linolenic acid; LA: linoleic acid; LACS: long-chain acyl-CoA synthetase; SDA: stearidonic acid; WS: wax synthase; VLC acyl-CoA: very-long-chain acyl-CoA

WEs are neutral lipids with excellent lubricating properties and are therefore of considerable industrial interest. In many organisms, WE biosynthesis proceeds through a relatively simple two-step pathway (Wenning et al. 2017). First, a fatty acyl reductase (FAR) converts an activated fatty acyl precursor, typically acyl-CoA or acyl-ACP, into the corresponding fatty alcohol. Subsequently, a wax synthase (WS) transfers the acyl group from acyl-CoA to the hydroxyl group of the fatty alcohol, generating a WE (Fig. 2). However, this simplified scheme does not apply universally, and variations in enzymes, substrates, and intermediates occur among organisms (Hofvander et al. 2011; Röttig and Steinbüchel 2013).

Historically, spermaceti oil derived from the sperm whale (*Physeter macrocephalus)* represented an important natural source of WEs for high-performance lubrication. Following the international ban on sperm whale hunting, this source became unavailable. Although chemical synthesis of WEs is feasible, the process remains expensive, which has limited broader industrial use. In this context, plant-based WE production has emerged as a potentially sustainable and economically alternative (Durrett et al. 2008). Substantial progress has made in reconstructing jojoba-derived WE biosynthetic pathways in heterologous plant hosts, particularly in *A. thaliana* and several oilseed crops. Expression of *FAR* and *WS* genes from jojoba, either alone or in combination with *FAE1* from different species, enabled the production of WEs up at levels reaching up to 60% of seed oil in some transgenic lines (Lardizabal et al. 2000; Zhu et al. 2016; Ivarson et al. 2017). In *A. thaliana*, heterologous WS has been demonstrated for both common and tailored molecular species, including long-chain monounsaturated WEs such as oleoyl-oleate (Heilmann et al. 2012). Subsequent studies have focused on improving WE yield and tailoring product composition through enzyme selection, subcellular targeting, and host metabolic modification. By selecting efficient enzyme combinations and modulating host FA composition, Iven et al. (2016) achieved high WE accumulation in Camelina seeds, producing oleoyl ester-type WEs at levels exceeding 60% of seed oil. Ruiz-Lopez et al. (2017) further showed that combining FAR and WS enzymes from different sources with additional FA-modifying enzymes, such as thioesterases, enabled the production of whale oil-like WEs in Camelina seeds. Similarly, Zhu et al. (2016) demonstrated that three Brassicaceae oilseed crops, *Crambe abyssinica*, *B. carinata*, and *C. sativa,* could be engineered to as accumulate up to 30% WEs in seed oil by expressing jojoba WE biosynthetic genes together with FA-regulatory activities, with *C. abyssinica* showing particularly favorable performance (Zhu et al. 2016).

Further optimization of WE biosynthesis has been achieved through enzyme selection, subcellular targeting, fusion protein design, and manipulation of endogenous FA metabolism. In *A. thaliana* seeds, initial co-expression of the mouse-derived enzymes *MmFAR1* and *MmWS* resulted in WE accumulation of 22 mg/g seed dry weight, predominantly comprising molecular species such as 20:1–18:2 and 18:1–18:1 (Heilmann et al. 2012). Targeting these enzymes to lipid droplets through fusion with oleosin (*Oleo3*) increased WE accumulation to 45 mg/g seed, while also altering product composition (Heilmann et al. 2012). Higher yields were subsequently achieved by combining the bacterial fatty acyl reductase *MaFAR* from *Marinobacter aquaeolei* with the jojoba *ScWS*, which enabled WE production of up to 108 mg/g seed, with oleyl gondoate (18:1–20:1) and 20:1–20:1 as the major products (Iven et al. 2016). A simplified strategy using a ScWS-MaFAR fusion protein produced 23 mg/g seed, and co-expression of this fusion construct with *MaFAR* further increased accumulation to 64 mg/g seed (Yu et al. 2018). In parallel, modification of endogenous FA metabolism enabled compositional tailoring. Expression of *Oleo3-MmFAR1* and *Oleo3-MmWS* in the *fae1fad2* mutant background reduced total yield but increased the proportion of oleyl oleate (18:1–18:1) to 65 mol% of total WEs, demonstrating the feasibility of engineering product specificity (Heilmann et al. 2012). For the production of very-long-chain WEs, co-expression of jojoba *ScFAR*, *ScWS*, and the FA elongase *ScFAE1* in *C. abyssinica* generated species such as 22:1–20:1, 22:1–22:1, and 24:1–22:1, accounting for up to 18% of total seed oil in advanced generations (Zhu et al. 2016; Li et al. 2019).

In addition to seed-based systems, WE biosynthesis has also been successfully targeted to vegetative tissues. Transient co-expression of chloroplast-targeted *MaFAR* and *AtPES2* in *N. benthamiana* leaves produced WEs containing medium-chain acyl groups such as 12:0 and 14:0 at approximately 0.9% of leaf dry weight (Aslan et al. 2014). Together, these studies highlight the versatility of plant hosts for both quantitative enhancement and compositional customization of WE biosynthesis.

The successful engineering of WEs in model plants and oilseed crops suggests that major cereals such as rice, maize, and wheat could also serve as platforms for WE production. Their large-scale cultivation and established transformation systems make them attractive candidates for industrial lipid biosynthesis. In principle, introduction of efficient *FAR* and *WS* combinations, together with tissue-specific promoters and supporting FA-modifying enzymes, could redirect endogenous FA metabolism toward WE accumulation in cereal tissues. Targeting synthesis to non-edible tissues such as leaves or stems may be particularly advantageous because it would minimize competition with food and feed uses. However, diversion of carbon flux away from starch accumulation may impair seed development, yield, and overall agronomic performance. In addition, stable multigene expression in cereals remains technically challenging, and regulatory approval will require careful evaluation, particularly when edible tissues are modified. Thus, although WE production in cereals appears feasible, its practical implementation will require precise pathway engineering, tissue-targeted expression, and rigorous assessment of agronomic and regulatory outcomes.

### Production of insect sex pheromones

In insects, particularly Lepidoptera, sex pheromones mediate species-specific communication and mating. The first chemically identified insect sex pheromone was bombykol (E,Z)-10,12-hexadecadien-1-ol, isolated from the silkworm moth *Bombyx mori* (Löfstedt and Xia 2021). Since then, sex pheromones have been identified in more than 600 lepidopteran species, many of them agriculturally important pests (Petkevicius et al. 2020). Approximately 75% of lepidopteran sex pheromones are fatty alcohols, aldehydes, and acetate esters with chain lengths of C_10_–C_18_ and one to three double bonds (Ando et al. 2004). Their biosynthesis generally begins with palmitoyl-CoA, which undergoes desaturation, chain shortening through β-oxidation, or elongation, followed by conversion of the resulting fatty acyl intermediates into alcohols, aldehydes, or acetates by fatty acyl-CoA reductases and acetyltransferases (Fig. 2). The coordinated activities of these enzymes generate the species-specific pheromone blends required for signaling fidelity and reproductive isolation (Ding and Löfstedt 2015; Petkevicius et al. 2020).

Plant-based heterologous expression systems have emerged as promising platforms for the production of insect pheromone precursors. In this approach, plants are engineered to synthesize FA-derived pheromone intermediates, which can then be extracted and, when necessary, chemically converted into active pheromone compounds. Such systems offer a potentially scalable and environmentally friendly alternative to conventional chemical synthesis and may support applications such as mass trapping and mating disruption in integrated pest management (Sparks and Nauen 2015; Deutsch et al. 2018; Petkevicius et al. 2020). Early studies established the feasibility of this concept. Nešněrová et al. reported the first functional expression of a lepidopteran desaturase in plants by introducing an insect Δ11-desaturase into *Nicotiana tabacum*, leading to the accumulation of the pheromone precursor (Z)-11-hexadecenoic acid (or its methyl ester) in transgenic tobacco. This precursor was then chemically converted ex planta to the sex pheromone component (Z)-11-hexadecenyl acetate (Z11-16:OAc) via semi-synthesis. Field trials showed the acetate derived from the plant-produced precursor effectively attracting the cabbage moth *Mamestra brassicae*, confirming the biological activity of the plant-derived product (Nešněrová et al. 2004). Subsequent work expanded plant-based pheromone biosynthesis in *N. benthamiana*, where transit expression of up to four genes encoding consecutive biosynthetic steps enabled the production of moth pheromone components (Ding et al. 2014). Stable production systems have also been developed in oilseed hosts. In *C. sativa*, Xia et al. generated transgenic lines that accumulated the codlemone precursors (E)-9-dodecenoic acid and (E,E)-8,10-dodecadienoic acid, highlighting the suitability of this species as a stable production platform (Xia et al. 2021). In a related study, engineered *Camelina* expressing a palmitoyl-ACP-specific thioesterase and a Δ11-desaturase accumulated Z11-16:acid at more than 20% of total seed FAs, corresponding to approximately 40 g/kg seed. When these precursors were chemically converted into the corresponding alcohol, aldehyde, and acetate derivatives, the resulting blend showed field efficacy against the diamondback moth *Plutella xylostella* comparable to that of the chemically synthesized pheromone mixture (Ortiz et al. 2020; Wang et al. 2022). Similar production of pheromone precursors has also been demonstrated in *N. benthamiana* (Demski et al. 2022). More recent advances in synthetic biology have further improved the tunability and efficiency of plant-based pheromone production. Kallam et al. used copper-inducible dCas9EV2.1 synthetic transcriptional activators together with optimized multigene construct architecture in *N. benthamiana* to achieve tunable, high-yield de novo biosynthesis of the pheromone components (Z)-11-hexadecenol and (Z)-11-hexadecenyl acetate (Kallam et al. 2023). These results demonstrate that plants can function not only as passive production hosts but also as programmable chassis for the controlled synthesis of pheromone molecules and their precursors. Major cereals such as rice, maize, and wheat may also represent future platforms for the heterologous production of pheromone precursors and related lipid-derived compounds. However, most cereal studies to date have focused on increasing endogenous lipid accumulation rather than reconstructing complete pheromone biosynthetic pathways. As a result, pheromone production in cereals remains largely prospective. Several insect pheromone precursors have been successfully synthesized in model plant systems, hinting at a promising avenue for advancing this field. Integrating these findings with cereal crops could pave the way for creating transgenic varieties that not only enhance pest management through pheromone emission but also maintain agronomic performance. Moreover, leveraging advancements in synthetic biology, such as efficient multigene stacking strategies, could enable the establishment of complete pheromone biosynthetic pathways in cereals. This approach would necessitate careful optimization of tissue-specific gene expression to ensure that pheromone production occurs in targeted plant tissues, thereby maximizing the efficacy while minimizing potential trade-offs in plant growth and yield. Additionally, researchers could investigate the optimization of carbon partitioning within the plants to support the energy demands of the heterologous pathways. Insights from metabolic engineering could reveal ways to redirect carbon fluxes towards pheromone synthesis without detracting from essential metabolic processes that sustain plant health. Future exploration may also involve establishing field trials to evaluate the ecological impact and effectiveness of pheromone-producing cereal crops in real agricultural systems. By investigating these aspects, we can better assess the practicality and sustainability of using cereals for pheromone production in integrated pest management strategies. In conclusion, while the path to pheromone synthesis in major cereals remains largely prospective, the potential benefits for sustainable agriculture are significant. Continued research efforts will be critical in overcoming the existing challenges and unlocking the full capabilities of cereals as platforms for pheromone production.

## Challenges and future perspectives

Synthetic biology-driven metabolic engineering has established the design-build-test-learn (DBTL) cycle as a central framework for iterative optimization (Carbonell et al. 2018). The plant DBTL cycle is an engineering cycle of designing genetic circuits or metabolic pathways, building them into plant chassis via transformation or editing, testing their function through phenotyping and multi-omics, and learning from the data to refine subsequent designs (Wang and Demirer 2023; Park et al. 2023). This strategy is increasingly being applied to plant metabolic systems, including lipid biosynthesis in major cereals. Although cereals naturally accumulate only low levels of storage lipids, they possess intact lipid biosynthetic pathways that provide a foundation for metabolic intervention (Vanhercke et al. 2019; Chang et al. 2024). Targeted engineering strategies, including overexpression of endogenous enzymes, introduction of heterologous genes, and multigene stacking, have already increased oil content and modified lipid composition in crops such as rice and maize (Vanhercke et al. 2019; Liu et al. 2024a, b). For example, in rice, endosperm-specific expression of Arabidopsis *DGAT1* under the *Glb1* promoter, combined with knockout of *AGPL2,* which encodes a rate-limiting enzyme of starch biosynthesis, and *MTSSB1,* a regulator of aleurone layer thickness, increased grain oil content fivefold, from 2.33% to 11.72%, in the elite cultivar NG46 (Liu et al. 2024a, b). Given their extensive global cultivation, cereals represent attractive targets for the production of high-value lipids, including PUFAs, WEs and insect pheromones, with potential applications in nutrition, sustainable industry, and environmentally friendly agriculture. Importantly, cereals are central to global food security and their use as metabolic engineering platforms has long been constrained by both technical and practical limitations. Technical limitations include limited transformation efficiency, incomplete understanding of tissue-specific lipid regulation, and potential trade-offs with agronomic traits such as yield and grain quality (Assefa et al. 2025). Beyond these scientific hurdles, the strict regulation of genetically modified (GM) seeds has historically acted as a major bottleneck, slowing the transition of engineered crops from the laboratory to commercial markets due to exhaustive safety and environmental demands (Liang et al. 2025; Liu et al. 2025a, b). Although the commercialization of genetically modified crops has long been constrained by stringent regulatory oversight, recent developments suggest a gradual shift toward more enabling regulatory frameworks, creating new opportunities for the practical application of agricultural biotechnology (Liang et al. 2025). This evolving landscape may facilitate the use of cereals as scalable platforms for high-value lipid production and enhanced food security through metabolic reprogramming while ensuring comprehensive safety.

### Technical bottlenecks and outstanding challenges

Despite recent progress, current understanding of lipid biosynthetic genes and their regulatory networks in cereals remains incomplete. Although many core enzymes involved in lipid biosynthesis have been identified, pathway coordination, and tissue-specific lipid deposition in cereals are still not fully resolved. This knowledge gap limits the rational design of engineering strategies, particularly because enhancement of lipid content or modification of FA composition often requires simultaneous manipulation of multiple genes and regulatory components (Mäenpää et al., 1999; Zhu et al. 2020; Wu et al., 2024). A second major challenge is the species- and genotype-dependence of cereal transformation systems. Compared with model dicots such as *A. thaliana* and *N. benthamiana*, many monocot crops remain relatively recalcitrant to efficient genetic transformation. Although cereals have benefited from substantial methodological advances, transformation efficiency, transgene stability and genotype compatibility continue to constrain both basic research and practical applications. These limitations become even more significant when complex multigene lipid pathways must be introduced and coordinately expressed. In addition, the introduction of heterologous metabolic modules or strong redirection of endogenous carbon flux may impose a substantial metabolic burden on the host plant. Such perturbations can lead to unintended pleiotropic effects, including impaired growth, altered seed development, reduced fertility, or compromised grain quality (Assefa et al. 2025). Balancing lipid accumulation with normal plant development therefore remains a central challenge in cereal metabolic engineering. Finally, long-term biosafety, ecological, and regulatory considerations must be addressed before engineered cereals can be deployed widely. Potential gene flow from transgenic cereals to wild relatives, unintended ecological effects, and concerns regarding food and feed safety require careful long-term evaluation (Mangla et al. 2025).

### Priorities for future research

Future research should focus on addressing these constraints through the integration of advanced biotechnologies, systems biology and iterative engineering approaches. A key priority is to clarify the regulatory architecture of lipid metabolism in cereals by using multi-omics strategies, including transcriptomics, proteomics, metabolomics, and flux analysis. Such approaches will facilitate identification of key regulator genes, rate-limiting steps, and tissue specific promoters that can be exploited for precise metabolic reprogramming (Barthole et al. 2012). Another important priority is the identification and optimization of heterologous enzymes and synthetic gene modules with improved activity, specificity, and compatibility in cereal hosts. Once effective genetic components have been identified, iterative optimization through the DBTL cycle can be used to refine pathway performance and improve the accumulation of desired lipid products. In parallel, promoter engineering and tissue-specific expression strategies should be further developed to confine heterologous pathways to selected tissues or developmental stages, thereby minimizing negative effects on growth and agronomic performance (Zhu et al. 2020). The large biomass and agronomic scale of cereals also create opportunities for functional specialization of different tissues. Seeds may be used for the production of nutritionally enhanced lipids, whereas vegetative tissues could be engineered for the synthesis of industrial lipids or insect pheromone precursors, potentially including controlled release systems for improved pest resistance (Vanhercke et al. 2019; Izadi-Darbandi et al. 2020). Such tissue-partitioned engineering strategies may improve overall metabolic efficiency while reducing competition with essential grain storage functions. Continued improvement of cereal transformation and genome-editing systems will also be essential for expanding the range of tractable cereal chassis. Recent progress, such as the development of an efficient transformation system based on wheat haploid immature embryos, highlights the potential for new tools to accelerate genome editing and transgene integration in previously recalcitrant crops (Han et al. 2025). More broadly, modular and adaptable engineering strategies will be needed to support coordinated optimization of both nutritional and industrial lipid traits across diverse cereal species. Finally, sustainable deployment frameworks must be established alongside technical innovation. Long-term field trials, ecological risk assessment, food and feed safety evaluation, and transparent regulatory pathways will be necessary to ensure that engineered cereals can be developed responsibly and accepted broadly. Together, these priorities provide a roadmap for advancing cereals as versatile platforms for the production of high-value lipid for nutritional and industrial applications.

## Conclusions

Advances in plant lipid biology, metabolic engineering, and genome editing technologies have substantially improved the prospects for producing high-value lipid compounds in crop systems. Increasing knowledge of FA biosynthesis, TAG assembly, and seed oil regulation has enabled more precise strategies for redirecting carbon flux and introducing novel biosynthetic pathways. These developments have supported the production of diverse lipid-derived compounds, including EPA and DHA, WEs, and insect sex pheromones, demonstrating the versatility of engineered plants as biosynthetic platforms.

Among potential hosts, cereals offer particular advantages because of their agronomic scalability, established transformation and breeding pipelines, and importance in global agriculture. However, further progress is still needed to improve pathway efficiency, product accumulation, tissue specificity, and genetic stability, while also addressing translational and regulatory challenges. From a techno-economic perspective, cereal platforms offer both opportunities and constraints for high-value lipid biomanufacturing. Their large cultivation scale, high biomass productivity, relatively low-cost field production, and established harvesting, storage, and processing infrastructure may reduce upstream production costs once stable high-producing lines are developed. Compared with microbial fermentation, cereals rely on photosynthetic carbon fixation and can be scaled through existing agricultural systems, but they have longer development cycles, lower process controllability, greater environmental variability, and more complex regulatory requirements, especially when edible seeds are engineered. Compared with chemical synthesis, cereal-based biosynthesis may be advantageous for complex lipid molecules that require multi-step, regioselective, or stereoselective reactions, but product accumulation, tissue specificity, extraction efficiency, and batch-to-batch consistency must reach economically viable levels. Therefore, cereal platforms are most promising when target lipids have high market value, can be accumulated at sufficient levels in defined tissues, and can be produced without compromising grain yield, grain quality, or food-security functions.

Overall, the integration of metabolic engineering with modern synthetic biology provides a strong foundation for developing cereals as sustainable plant-based factories for high-value lipids, with promising applications in agriculture, nutrition, and the emerging bio-based economy.

## Data Availability

No new data were created or analyzed in this study. Data sharing is not applicable to this article.
